# A Validated LC-MS/MS Method for Performing Belatacept Drug Monitoring in Renal Transplantation

**DOI:** 10.3390/biomedicines11112955

**Published:** 2023-11-01

**Authors:** Stéphanie Chhun, Mathieu Trauchessec, Sophie Melicine, Frédéric Nicolas, Agathe Miele, Srboljub Lukic, Estelle Vilain, Lucile Amrouche, Dorothée Lebert, Dany Anglicheau, Eric Tartour, Julien Zuber

**Affiliations:** 1 Laboratory of Immunology, Georges Pompidou European Hospital and Necker Hospital, AP-HP, F-75015 Paris, Franceeric.tartour@aphp.fr (E.T.); 2 Faculty of Medecine, Université Paris-Cité, F-75006 Paris, France; dany.anglicheau@aphp.fr (D.A.); julien.zuber@aphp.fr (J.Z.); 3 Promise Proteomics, F-38040 Grenoble, Francedorothee.lebert@promise-proteomics.com (D.L.); 4 Department of Kidney and Metabolic Diseases, Transplantation and Clinical Immunology, Necker Hospital, AP-HP, F-75015 Paris, Francelucile.amrouche@aphp.fr (L.A.)

**Keywords:** belatacept, CTLA4-Ig, transplantation, kidney, pharmacokinetic

## Abstract

Belatacept, a CTLA4-Ig, was designed to prevent rejection and graft loss in kidney transplant recipients. This immunotherapy showed a long-term clinical benefit mainly on renal function and better glycemic control but was also associated with a higher number of severe infectious diseases, particularly CMV disease, and lymphoproliferative disease. Therapeutic drug monitoring usually guides the benefit–risk assessment of long-term immunosuppression. In this study, an analytical method by LC-MS/MS was developed in 20 microL of plasma for the belatacept quantification. Intra- and inter-assay precision and accuracy were lower than 20% for the limit of quantification, and 15% for higher concentrations. The method was implemented in our lab and provided data about the inter-variability (N = 108) and intra-variability (N = 33) of belatacept concentrations in kidney transplant recipients with a stable renal function, after conversion from a CNI- to a belatacept-based regimen.

## 1. Introduction

Belatacept is a second-generation fusion protein consisting of the extracellular domain of human Cytotoxic T-Lymphocyte Antigen 4 protein (CTLA4/CD28) linked to the Fc domain of human IgG1 (CTLA-4-Ig). Belatacept is used in calcineurin inhibitor (CNI)-sparing immunosuppressive regimens to help prevent rejection and graft loss, while also avoiding drug-induced nephrotoxicity in kidney transplant recipients. Belatacept inhibits the CD28-dependent costimulatory pathway in T cells through high-affinity binding to CD80/CD86 on antigen presenting cells [1].

Nowadays, belatacept has not gained much traction in routine clinical practice due to several factors: first, economic reasons; the lack of long-term experience with this drug, which leads physicians to fear early acute rejections and posttransplant lymphoproliferative disorders, especially in EBV-naive patients; and finally the need for intravenous administration. However, belatacept may present several advantages compared to CNIs in renal transplantation [2].

Notably, several studies have shown the indisputable clinical benefits of belatacept over CNIs, which have long been considered as the standard of care in terms of renal function, glycemic control, and related cardiovascular risk in kidney transplant recipients. Moreover, belatacept may also curtail de novo sensitization against donor HLA after kidney transplantation; this effect is most likely linked to its CTLA4/CD28 part. Indeed, functional CD28 co-stimulation is known to play a key role in germinal center reaction [3,4].

This function is supported by the compelling observation that belatacept-treated patients fail to mount a vaccinal response [5,6]. In relation to this reduced immune competence, belatacept has also been associated with an increased number of severe infectious diseases, particularly CMV disease, and lymphoproliferative disease. The incidence of these diseases is generally lower with CNI-based regimens [7,8,9,10].

It cannot be excluded that belatacept-related infectious complications result, at least in part, from drug overexposure. Indeed, Borni-Duval et al. [11] identified risk factors associated with BKV infections in kidney transplant recipients. Among them, they found mycophenolic acid overexposure at 3 months post-transplantation, of more than 50 mg.h/L, expressed as area under the curve, and through concentration of tacrolimus above 10 µg/L. Asberg et al. [12] also showed that 17 out of 19 (89.5%) patients with lower concentrations of calcineurin inhibitors, defined as trough ciclosporin concentrations below 150 μg/L or trough tacrolimus concentrations below 5 μg/L, showed DNAemia eradication, as compared with 73 out of 118 (61.9%) patients with higher concentrations.

Belatacept, unlike other immunosuppressants, is currently administered as a weight-based dose, with no monitoring for drug exposure or potential inter-individual variability. In addition, no assay exists to routinely assess belatacept concentrations in the clinic, and we lack extensive real-world pharmacokinetic data.

TDM is the cornerstone for CNI exposure monitoring, and is extensively used to guide the benefit–risk assessment of long-term immunosuppression and to refine the therapeutic window [13]. For this study, we set up a new mass spectrometry-based assay to make therapeutic drug monitoring (TDM) available for belatacept. We hypothesized that such an assay could help improve the safety of belatacept and its efficacy profile in kidney transplant recipients. Indeed, compared to a study previously published [14], we present an improved liquid chromatography mass spectrometry (LC-MS) based approach, which consists of a ready-to-use CE-IVD kit. This validated approach was used to determine the variability of serum belatacept concentrations in kidney transplant recipients after a switch from a CNI- to a belatacept-based regimen.

## 2. Patients

All consenting kidney transplant recipients receiving maintenance belatacept doses were enrolled at the outpatient transplant clinic of Necker Hospital (Paris, France), between March and June 2022 (N = 108). To ensure that the steady-state concentration had been achieved, patients had to have been treated with belatacept for at least 3 months, receiving doses every 4 weeks, after an induction phase, during which tacrolimus was progressively tapered off. More specifically, belatacept was started at 5 mg/kg on days 0, 14, 28, 42, and 56, and then dosing was spaced out to a single monthly dose. The maintenance belatacept regimen also included an antiproliferative drug (mycophenolic acid or azathioprine) and oral steroids.

Blood samples were collected just before the belatacept administration and were centrifuged at 4000 trs/min at 20 °C in order to separate the plasma.

## 3. Materials and Methods

### 3.1. Reagents and Labware

The multiplex belatacept/abatacept mAbXmise kit was obtained from Promise Proteomics (Ref: GTDM, Promise Proteomics, Grenoble, France). mAbXmise is a ready-to-use, CE-IVD kit, enabling the quantification of both drugs using mass spectrometry and an internal standard which is a full-length stable isotopically labelled version of abatacept, which also works perfectly for quantifying belatacept as both drugs’ sequences differ only by 2 amino acids. To make the kit easy to use in a clinical laboratory, all the reagents and consumables used to prepare plasma samples for LC-MS injection were provided. The calibration standards and quality controls were prepared by spiking drug-free human plasma—obtained from the French national blood service (EFS, Grenoble, France)—with commercial belatacept (Nulojix^®^, Bristol Myers Squibb, New York, NY, USA). LC-MS/MS-grade acetonitrile was purchased from Merck-Sigma (St. Louis, MO, USA), LC-MS-grade water and formic acid were from Fisher Chemicals (Illkirch, France).

### 3.2. Preparation of Calibration Curves and Internal Quality Controls

Calibrators and quality controls (QC) were provided in the kit: [0, 1, 5, 10, 20, and 100] µg/mL for calibrators, and [3, 25] µg/mL for QCs. Calibrators and QCs were prepared from independent stock solutions of both plasma and therapeutic mAbs. In order to perform the validation of the analytical performances of the kit, one additional calibrator [50 µg/mL] (CALsup), and two supplementary QCs [1, 75] µg/mL (LLOQ and highQC, respectively) were prepared. Calibrators and QC were prepared as described previously [15].

Samples were prepared according to the instruction manual provided, and as previously described [15,16]. Briefly, 20 µL of sample (calibration standard, QC, or test plasma) was loaded into wells on the mAbXmise plate, containing the internal standard lyophilized at the bottom of each well, and diluted with 80 µL of Buffer A from the kit.

Plates were incubated for 1 h at room temperature with agitation. Then, the global IgG content (endogenous IgG pool, belatacept drug and stable labelled belatacept-like standard) was extracted by immunocapture on the PuriXmise plate. Then, samples were eluted before drying in a speed-vacuum (Martin Christ, Osterode am Harz, Germany). After re-solubilization, samples were digested using CutXmise enzyme overnight at 37 °C. The next day, CutXStop was added to stop digestion before injection of 20 µL of sample into the LC-MS/MS system.

### 3.3. LC-MS/MS Conditions and Instrumentation for Development and Validation

Proteotypic peptides for belatacept were selected as previously described [17]. The final MRM transitions selected are listed in Table 1.

The chromatographic system used was an Exion system with binary pumps (Sciex, Framingham, MA, USA), the autosampler temperature and the column oven were set, respectively, to 15 °C and 40 °C. Chromatographic separation of peptides was achieved on a BioZen™ 2.6-µm Peptide XB-C18 LC column measuring 100 × 2.1 mm (Phenomenex, Torrance, CA, USA). The mobile phases were a mix of 0.1% formic acid in water (A) and 0.1% formic acid in acetonitrile (B). To achieve chromatographic separation, the elution gradient was as follows: 95% phase A from 0 to 2 min, decrease to 90% phase A from 2 to 2.1 min, decrease to 75% phase A from 2.1 to 5 min, decrease to 10% phase A from 5 to 5.1 min, flush with 10% phase A from 5.1 to 8 min, return to 95% phase A from 8 to 8.1 min, and stabilize with 95% phase A up to 10 min. The flow rate was 300 µL/min throughout the run, except between 5.1 and 8 min, when the flow rate was 500 µL/min. The mass spectrometer used was a triple-quadrupole 6500 QTRAP (Sciex, Framingham, MA, USA). Source parameters were: curtain gas 30 psi, Ionspray voltage 5500 V, and source temperature 550 °C. Ion source gas 1 was applied at 40 psi, and the pressure for ion source gas 2 was set to 45 psi. Declustering potential was set as variable, inlet potential and collision cell exit potential were set to 10 and 13, respectively.

### 3.4. Method Validation

The method was fully validated according to the international guidelines published when the experiments were performed (European Medicines Agency 2011; Food and Drug Administration 2018) and the validated parameters were consistent with those set out by the French certification institution COFRAC for analytical methods used for diagnostic purposes in clinical labs in France. For the analytical validation of the method, six calibration points were used.

#### 3.4.1. Linearity and Lower Limit of Quantification

For the linearity assessment, double blank, zero samples and CAL samples (between 1 µg/mL and 100 µg/mL) were prepared in 6 replicates, and analyzed on 3 different days. Curves using the zero and six calibration standards (1, 5, 10, 20, 50, 100 µg/mL) were plotted. The ratio of the peak area of the analyte of interest over its corresponding IS was plotted against the nominal concentration of the analyte. A minimum of 75% of the standard calibration samples had to be within ±15% of the nominal concentration, except for the lower limit of quantification (LLOQ), which is defined as the lowest amount that can be precisely and accurately quantified within ±20%.

#### 3.4.2. Accuracy and Precision

Accuracy and precision were assessed based on the LLOQ (1 µg/mL) and three QCs corresponding to low (3 µg/mL), mid-range (25 µg/mL), and high (75 µg/mL) concentrations. Intra-day accuracy and precision were determined using six (*n* = 6) samples injected on the same day (day 1). Inter-day accuracy and precision were determined by injecting on 3 different days, 6 replicates of the following samples QClow (3 µg/mL), QCmid (25 µg/mL) and QChigh (75 µg/mL), and LLOQ samples (1 µg/mL). Precision was assessed by calculating the coefficient of variation (CV) between the replicates. Accuracy was reported as the relative difference between the concentration measured and the theoretical value. Accuracy and precision should be less than 15% for QCs, or 20% for LLOQ.

#### 3.4.3. Selectivity, Carry-Over and Matrix Effect

Specificity was investigated using “drug-free” plasma samples from six healthy individuals. Six double blanks (processed matrix sample without analyte and without IS) and 6 LLOQ samples (drug-free matrix spiked with belatacept at 1 µg/mL before processing) were analyzed.

Carry-over was estimated by injecting the highest calibration standard (100 µg/mL), immediately followed by a blank sample (mobile phase A). The peak area measured at the retention time for analytes in the blank sample should be less than 20% of the area measured in the LLOQ sample for analytes, and 5% of the area measured for the IS.

Matrix effects were investigated using 6 batches of 4 types of specimens (citrated plasma, heparinized plasma, EDTA plasma, and serum) from independent donors. Belatacept (therapeutic) was spiked either into these matrices or into solvent at low (3 µg/mL) and high (75 µg/mL) QC concentrations. Samples were then prepared and digested as above. Each sample was prepared in triplicate. Then, for each analyte and the IS, the matrix factor (MF) was determined by calculating the ratio of the peak area in the presence of plasma to the peak area in solvent. The IS-normalized MF was calculated by dividing the MF for the analyte by the MF for the IS.

### 3.5. Statistical Analysis

All continuous variables were expressed as means ± standard deviation. The interindividual variability was expressed as a coefficient variation of all trough concentrations, expressed as a percentage (CV%). The intraindividual variability was determined in patients who had trough concentrations during 3 consecutive visits. All statistical analyses were performed using Graph-Pad Prism 9.0 software (GraphPad Software, San Diego, CA, USA). This section may be divided by subheadings. It should provide a concise and precise description of the experimental results, their interpretation, as well as the experimental conclusions that can be drawn.

## 4. Results

### 4.1. Chromatograms

**Figure 1:**
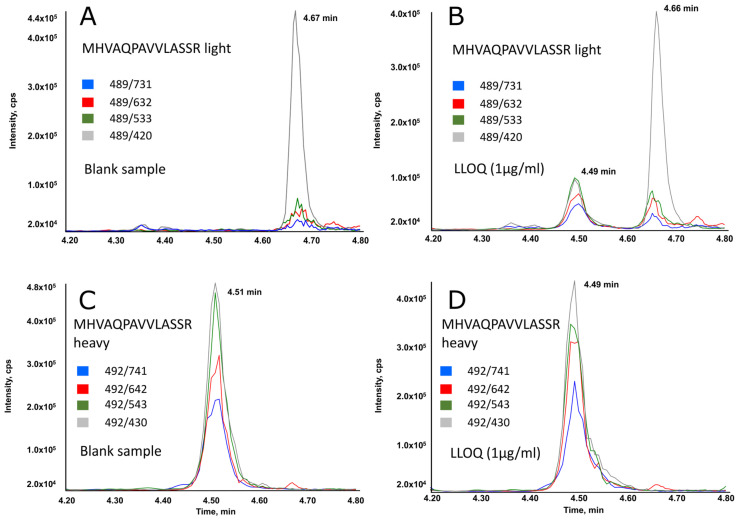
Extracted ion chromatograms obtained for (**A**) light peptide monitored in a blank sample (plasma without therapeutic belatacept) prepared according to the mabXmise kit, thus containing the internal heavy standard; (**B**) heavy peptide monitored in a blank sample; (**C**) light peptide monitored in sample spiked at 1 µg/mL with therapeutic belatacept and prepared according to the mabXmise kit, thus containing the internal heavy standard; (**D**) heavy peptide monitored in sample spiked at 1 µg/mL with therapeutic belatacept. It can be noticed that the light peptide was not detected at expected RT (4.5 min) in the blank sample while it was detected in the sample at 1 µg/mL. MS approach specificity allows for the separating interference of light peptide detected at 4.6 min.

Figure 1 displays typical chromatographic profiles (selected reaction monitoring mode) for a blank plasma sample (A,C), and for sample at the LLOQ (B,D). As shown, the peptide specific for belatacept was adequately separated from any potentially interfering peaks. The unlabeled peptide co-eluted perfectly with its labeled analog.

### 4.2. Method Validation

We validated the GTDM mAbXmise method according to the criteria set out in the international guidelines [18,19]—see Table 2.

#### 4.2.1. Limit of Quantification and Linearity

Linearity of the method was determined over the calibration range based on linear regression. Calibration curves were linear for belatacept peptides detected from samples containing concentrations ranging from 1 to 100 µg/mL, with r^2^ ≥ 0.99. The back-calculated concentrations of the calibrators were within ±15% of the expected value, what is compliant with required specifications.

Slopes, intercepts, and coefficients of determination obtained for mean values and standard deviations were as follows (*n* = 18): y = 0.11742x + 0.01052 and r^2^ = 0.99329 for pep_ MHVAQPAVVLASSR, where x is the concentration in µg/mL and y is the area ratio.

#### 4.2.2. Accuracy and Precision

Table 2 describes the evaluation of accuracy and precision for the samples tested. For parameters evaluated (within-run accuracy and precision and between-run accuracy and precision), bias and RSD were <20% for LLOQ and <15% for the three QC samples analyzed.

#### 4.2.3. Selectivity, Carry-Over, and Matrix Effect

According to Table 2, selectivity and the matrix effect met the acceptance criteria set out in the guidelines. For carry-over, with the gradient described a residual signal corresponding to the unlabeled peptide (analyte) with an intensity above the cutoff of 20% of LLOQ was observed in the blank injected just after the highest calibration point. In the second blank, the signal was less than 20% of the LLOQ value. It is thus advised to add a blank sample after the highest calibration point (100 µg/mL). No washout is required after the 50 µg/mL calibration point. This carryover is system- and column-dependent, it should be checked by the validation method described. Indeed, in other labs, no carry-over was detected.

### 4.3. Clinical Sample Analyses: Determination of the Inter-Individual Variability and Intra-Individual Variability of Belatacept Concentrations in Kidney Transplant Recipients

**Figure 2:**
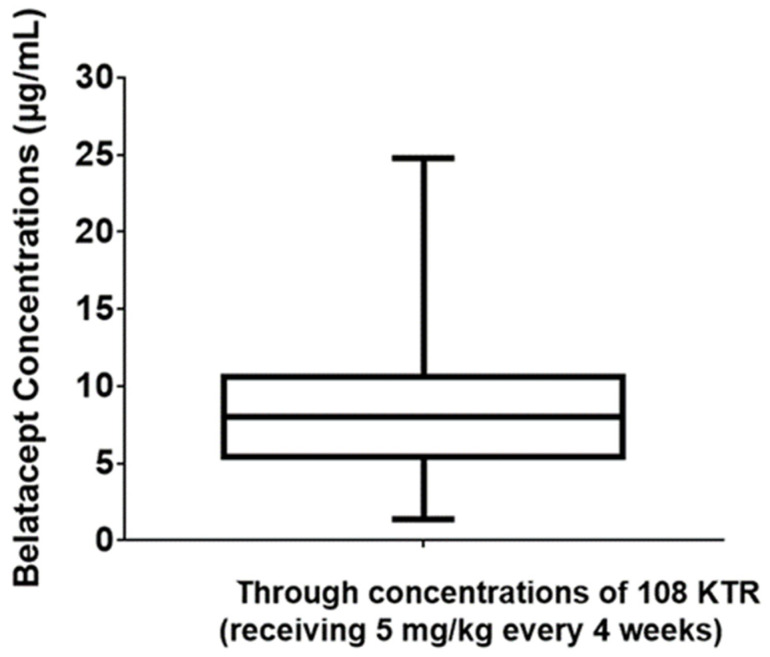
Observed belatacept trough concentrations and inter-individual variability in 108 kidney transplant recipients.

**Figure 3:**
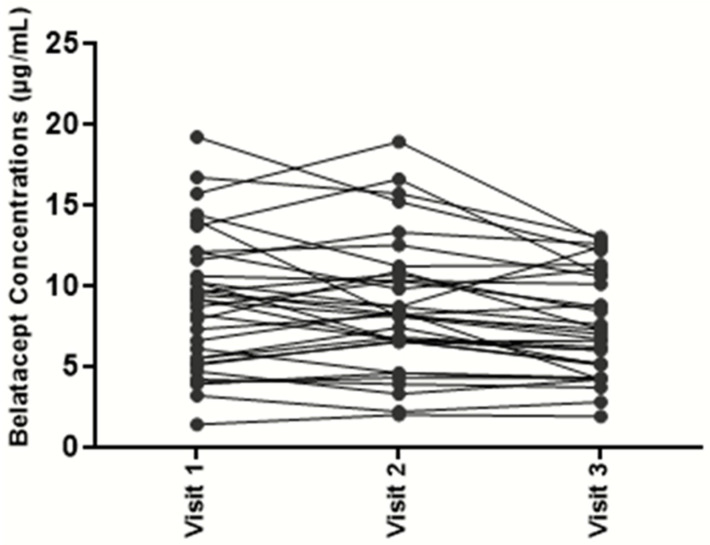
Observed belatacept trough concentrations in 33 kidney transplant recipients during 3 consecutive visits.

A total of 252 trough concentrations of belatacept, from 108 kidney transplant recipients (45% women, 25–85 years old) were measured. All patients displayed a stable graft function with a mean estimated glomerular filtration rate at 44 mL/min/m^2^, and had been treated with a maintenance regimen belatacept dose for at least 3 months. Plasma samples were used to assess steady-state belatacept concentrations. Belatacept trough concentrations were observed to vary considerably, from 1.4 to 24.8 µg/L, with a mean (±SD) concentration of 8.4 ± 3.9 µg/L (Figure 2). Based on these results, the mean inter-individual variability was estimated at 46%. To assess intra-individual variability, belatacept concentrations were assessed at 3 consecutive time points in 37 kidney transplant recipients. Mean intra-individual variability was only 17% (Figure 3).

## 5. Discussion

The aim of this study was to validate the use of a kit approach to quantify belatacept in human plasma using LC-MS/MS.

Although immunoassays are most widespread in clinical labs for quantification of therapeutic biomolecules, they also present discrepancies and can be long to develop due to the need of high affinity antibody capture reagents. For these reasons, we have chosen here to work with an alternative technology, which is mass spectrometry. Among the main advantages, mass spectrometry can detect and quantify biomolecules in complex matrices without requiring the use of capture antibodies, which makes it possible to quickly develop a quantitative assay and which is also economically competitive. Moreover, the technology presents an analytical robustness compared to immunoassays [20]. The robustness is reinforced in our approach by the use of internal standards which correct for variations in the patient samples and during the analytical process, as well as potential variability, which are operator-dependant or environment-dependant.

The kit format has facilitated the use of the assay in the lab and the implementation of the method, on the mass spectrometer already installed in our clinical lab, where it is used for many other assays. The therapeutic monitoring of belatacept concentrations was thus simplified, even for non-expert LC-MS users.

Compared to previous attempts to monitor therapeutic concentrations of belatacept, the use of a belatacept-like stable-isotope-labeled internal standard strengthens the robustness of our method, notably as it provides better correction for sample variability. The results showed all measured performance parameters to be within the requirements for bioanalytical method validation according to international agencies (i.e., European Medicines Agency, Food and Drug Administration, etc.).

The method was accurate enough to perform belatacept TDM in adult kidney transplant patients (mean intra-individual variability evaluated at 17%).

Transplant patients are currently treated with a weight-adjusted dosage of belatacept. However, our results revealed a high variability of belatacept levels between patients treated on a maintenance regimen (range 1.4–24.8 µg/L). As there are limited pharmacological options in transplantation, after switching to belatacept, this finding strongly supports the need for drug monitoring to assess the immunosuppressive burden in patients in an individualized manner. The relevance of such a strategy is supported by the low intra-patient variability observed. It is true, however, that our study does not demonstrate yet the clinical relevance of TDM for belatacept, it only focuses on the analytical feasibility demonstrated on an important cohort. To this end, further studies are needed. Pharmacokinetics plays an important role in optimizing drug dosing in a given population. Belatacept clearance rates and trough concentrations could be used to refine dosing. Currently, no large set of belatacept PK/PD data is available to define the target versus toxic trough concentrations. Our preliminary results lay the groundwork for large prospective studies addressing the issue of whether belatacept TDM correlates with clinical events, such as rates of infections, malignancies, or rejections. Clinical studies on pediatric patients would also be of importance in the future to see whether TDM can benefit this specific population.

## 6. Conclusions

Here we reported the development and the validation of an accurate and sensitive LC/MS-MS to quantify belatacept concentrations in plasma samples. This method can be used in clinical studies to conduct exposure-response analyses in order to optimize the immunosuppression treatment in kidney transplant recipients. Belatacept therapy has been broadly implemented in kidney transplant recipients, using a “one dose fits all” strategy. We believe, however, that the benefit/risk balance for belatacept could be better optimized, by implementing TDM. This strategy could be used to provide a more personalized approach, potentially alleviating the iatrogenic effects of life-long immunosuppression.

## Figures and Tables

**Table 1:** Surrogate peptides used to quantify belatacept in plasma, and their corresponding MRM transitions. All peptides listed were used as “quantifier” peptides.

Molecule	Peptide Sequence	Q1 m/z	Charge Parent Ion	Q3 m/z	Fragment Ion
Belatacept	MHVAQPAVVLASSR	489.27	3+	731.441	Y7 (quantifier)
				632.373	Y6 (quantifier)
				533.304	Y5 (quantifier)
				420.220	Y4 (quantifier)
	MHVAQPAVVLASS [13C6,15N4]R	792.60	3+	741.449	Y7 (quantifier)
				642.381	Y6 (quantifier)
				543.312	Y5 (quantifier)
				430.228	Y4 (quantifier)

**Table 2:** Summarizes analytical parameters evaluated and results obtained for each criteria.

Analytical Characteristic	Replicates per Run	Days	Expressed as	Sample	Expected Performance	Results
Specimen	3 (for 4 specimens: citrated plasma, EDTA plasma, heparinized plasma, and serum; 6 batches each)	1	Bias (%)	LowQC HighQC	<15% <15%	<15% <15%
		
Specificity (interferences)	3 (for 4 specimen types; 6 batches of each specimen)	1	Mean of DB/LLOQ (%) for the analyte Mean of DB/LLOQ (%) for the IS	Double Blank(DB) LLOQ	<20% <5%	12.1% 1.45%
Matrix effect	3 (for 4 specimen types; 6 batches of each specimen)	1	CV of normalized MF (%)	in matrix LowQC HighQC in PBS LowQC HighQC	<15%	LowQC 9.3% HighQC 6.5%
Within-run accuracy	6	1	Bias (%)	LLOQ LowQC MidQC HighQ	<20% <15% <15% <15%	6.7% 5.0% 0.1% 0.3%
Between-run accuracy	6	3	Bias (%)	LLOQ LowQC MidQC HighQC	<20% <15% <15% <15%	3.6% 2.0% 3.2% 5.0%
Within-run precision	6	1	RSD (%)	LLOQ LowQC MidQC HighQC	<20% <15% <15% <15%	6.7% 3.4% 7.1% 3.6%
Between-run precision	6	3	RSD (%)	LLOQ LowQC MidQC HighQC	<20% <15% <15% <15%	7.0% 6.8% 5.6% 4.6%
Linearity	6	3	R^2^	Kit calibrators CAL0, CAL1, CAL2, CAL3, CAL4, CAL5 Additional calibrators CAL sup	R^2^ ≥ 0.99 6 CAL 5 CAL <15% (<20% for CAL1)	0.99329 (6CAL) 0.99317 (5CAL) <15%
LLOQ	6	3	Mean LLOQ/CAL0 (%) for the analyte Bias (%) RSD (%)	LLOQ CAL0	LLOQ/CAL0 > 5% Bias < 20% RSD < 20%	LLOQ/CAL0 > 10% Bias: 3.6% RSD: 7%
Carry-over	3	3	Signal for unlabeled peptide relative to LLOQ (%) Signal for labeled peptide relative to signal for IS (%)	Blank after highest standard	<20% <5%	28.75% 0.29%
Metrology	3	1	Bias (%) RSD (%)	LowQC HighQC	Bias < 20% RSD < 20%	LowQC 0.0% 4.9% HighQC 7.0% 5.2%

Concentration values for CAL samples: CAL0 = 0 µg/mL, CAL1 = 1 µg/mL, CAL2 = 5 µg/mL, CAL3 = 10 µg/mL, CAL4 = 20 µg/mL, CAL5 = 100 µg/mL; concentrations of QC samples: LowQC = 3 µg/mL, MidQC = 25 µg/mL, HighQC = 75 µg/mL, LLOQ = 1 µg/mL, CALsup = 50 µg/mL.

## Data Availability

The analytical data presented in this study are available on request from the corresponding author. The clinical data are not publicly available due to privacy and ethical concerns.
